# Release kinetics of the model protein FITC-BSA from different polymer-coated bovine bone substitutes

**DOI:** 10.1186/s13005-019-0211-y

**Published:** 2019-11-11

**Authors:** Julian Lommen, Lara Schorn, Alexis Landers, Henrik Holtmann, Karin Berr, Norbert R. Kübler, Christoph Sproll, Majeed Rana, Rita Depprich

**Affiliations:** 1 Department of Oral and Maxillofacial Surgery, Heinrich-Heine-University, Moorenstraße 5, 40225 Düsseldorf, Germany; 2 Department of Oral and Maxillofacial Surgery, Evangelisches Krankenhaus Hattingen, Bredenscheider Straße 54, 45525 Hattingen, Germany; 3 Department of Oral and Maxillofacial Surgery, Malteser Clinic St. Johannes, Johannisstraße 21, 47198 Duisburg, Germany

**Keywords:** FITC-BSA, Polymer-coated, Collagen matrices, Bovine, Release kinetics

## Abstract

**Background:**

Controlled release of proteins bound to conventional bone substitutes is still insufficient. Therefore, this study evaluates in-vitro release kinetics of the model protein FITC-BSA (fluorescein conjugated bovine serum albumine) from insoluble bovine collagenous bone matrices (ICBM) with different polymer coatings. Analyzes aim at comparing FITC-BSA release from uncoated versus coated ICBM over time to find bone substitute coatings with consistent release profiles.

**Methods:**

Release kinetics of FITC-BSA from uncoated as well as coated ICBM with five different polymers (RESOMER R 203 H, RG 503 H, RG 504 H, RG 505, L 206 S) were measured over a period of 11 days (d). Measurements were conducted after 6 h (h), 12 h, 24 h, 3 d, 5 d, 7 d, 9 d and 11 d with six samples for each coated ICBM. Two groups were formed (1) with and (2) without medium change at times of measurement. For each group ANOVA with post-hoc Bonferroni testing was used. Scanning electron microscopy assessed morphologic differences between ICBM coating.

**Results:**

In group 1 approx. 70% of FITC-BSA release from uncoated ICBM occurred after 6 h compared to approx. 50% in group 2. Only polymers with medium inherent viscosity, i.e. RESOMER RG 503 H, constantly showed significantly more FITC-BSA release throughout 11 d than uncoated ICBM (*p* = 0.007). The same was found for group 2 (*p* = 0.005). No significant differences between PLA and PLGA polymers were found. Scanning electron microscopy results indicate a weak adhesion of polymer coatings to ICBM explaining its rather weak retentive effect on overall FITC-BSA release.

**Conclusions:**

Medium molecular size polymers reduce the overall released FITC-BSA from ICBM over time. In clinical practice these polymers may prove ideal for bone substitute materials.

## Background

Reconstructive surgery of osseous maxillo-facial structures often requires autologous or allogeneic bone replacement. Due to its osteogenetic, osteoinductive and osteoconductive properties autologous bone is still considered the gold standard bone graft. Disadvantages of autologous bone harvesting are its limited availability and under certain circumstances considerable donor-site morbidities perhaps with irreversible damage to patients [1]. Hence, conventional natural or synthetic bone substitute biomaterials have been implemented as volume-stable placeholders to enable in-growth of new endogenous bone tissue. In that regard, they promote cell adhesion processes which lead to cell proliferation and production of extracellular matrix necessary for bone formation [2]. Obviously, these biological pathways come up against their limits in great boney defects where a complete bone formation is not reached solely by guiding bone-generating cells. Therefore, conventional bone substitute materials have yet failed to fully engage in the dynamic tissue remodeling processes of bone. Considering this, modern tissue engineering of bone replacement materials aims at combining new carrier matrices with cells and cytokines (i.e. interleukin-1 [IL-1], interleukin-6 [IL-6] and tumor necrosis factor-α [TNF-α]) to best possibly mimic autologous bone [3]. Therefore, modern bone replacement materials should be biocompatible [4], biodegradable [5], porous [6] and mechanically resolute [7]. Insoluble bovine collagenous bone matrices (ICBM) as bone substitutes have been shown to meet the aforementioned requirements with collagen being biocompatible, biodegradable (up to six months) and porous (from 85 to > 325 μm) [8]. Smaller pores support cell adhesion, bigger pores cell migration and angiogenesis [9]. The ability to use ICBM in reconstruction of voluminous osseous maxillo-facial defects was shown [10]. Since serum concentrations of endogenous cytokines are insufficient to bridge critical-size-defects prepared with uncoated ICBM through induction of bone formation coating of ICBM with cytokines and growth-factors was introduced in 2002. Here, recombinant bone morphogenetic protein-2 (rhBMP-2), a strong initiating protein for bone regeneration, was combined with ICBM and approved by the American Food and Drug Administration (FDA) for medical treatment of vertebral bone defects (InFuse®, Medtronic, Memphis, Tennessee, USA) [11, 12]. Observed side effects such as inflammation, excessive bone formation and questionable carcinogenic potency were attributed to the uncontrolled release of cytokines and proteins from ICBM [13]. Because solutionized cytokines often show a short half-life it seemed that many ICBM are overloaded with cytokines to reach the desired bone regeneration [14]. Hence, the call for a controlled and controllable release of cytokines from ICBM was made. To date release kinetics of cytokines from collagen matrices are of high scientific interest [15]. Synthetic biodegradable polymers like polylactic acid (PLA) and polylactic glycolic acid (PLGA) have been used in various biomedical studies and products such as resorbable sutures, stents, screws, plates, drug carriers as well as tissue engineering to enhance the quality of drug carriers [16, 17]. Since the release kinetics of substances from polymer-coated ICBM depend on the physicochemical properties of the polymers, PLA and PGLA were designed with specific grades of hydrophilic, crystalline and degradation properties. Due to the linear relationship between fluorescence and concentration fluorescein isothiocyanate-labeled bovine serum albumin (FITC-BSA) is often used as a model protein for pharmacological release kinetics testing [18, 19].

Therefore, in this study we aimed at analyzing the suitability of PLA and PLGA polymer coatings to control the release kinetics of growth factors from ICBM using the model protein FITC-BSA.

## Methods

### Design of insoluble bovine collagenous bone matrices (ICBM)

A cuboid (1x1x0.5 cm) bovine collagen matrix was produced as a threedimensional scaffold on the basis of an already established patent [20] and procedure [21]. Fresh, approximately 1 cm thick bovine femur bone slices were stored at − 80° Celcius (°C) until further processing (Table 1). Only ICBM with medium cancellous bone density were used in the experiments.

**Table 1:** Production steps of insoluble bovine collagenous bone matrices ICBM

Step 1	Coarse degreasing of femur bone marrow with a steam jet
Step 2	Thorough degreasing of femur bone marrow by application of chloroform:methanol (3:1) solution for three consecutive times at 24 h, 72 h and 24 h intervals.
Step 3	Washing with distilled water, bleaching by application of hydrogen peroxide [H_2_O_2_] (3% for 15 min), washing with distilled water.
Step 4	Demineralization by application of 0.5 M (M) hydrogen chloride [HCL] for three consecutive times at 1.5 h intervals.
Step 5	Washing with distilled water, sawing out the cuboid femur slices, application of 0.5 M HCL for 1 h, washing with distilled water, inactivation of osteoinductive matrix proteins by application of 4 M guanidine-HCL/Tris*-HCL (pH value 7.0) for 24 h at 4 °C.
Step 6	Application of 50 mM Tris-HCL/150 mM sodium chloride [NaCl] (pH value 7.0), washing with distilled water for 30 min, temporary freezing at − 80 °C for 72 h, lyophilization at − 4 °C and 0.05 mbar for 24 h, storage at 4 °C until gamma sterilization and final sterile storage in 50 ml containers at room temperature (RT).
Step 7	Assorting of ICBM with medium cancellous bone density for use in this study.

* tris (hydroxymethyl)aminomethane

### FITC-BSA labeling and coating of ICBM

FITC-BSA was purchased in 5 mg packages from Invitrogen© (Molecular Probes©, Oregon, USA). LOT 1081751 was used for the main experiments. At concentrations between 5 and 500 μg/ml a linear relationship of FITC-BSA and the measured fluorescence values detected by the GENios© reader is given. Each ICBM was washed with distilled water (1 min.), centrifuged (1000 rpm for 3 s), labeled with 500 μg FITC-BSA and pinned to a needle to avoid premature release of FITC-BSA. The used polymer coatings are displayed in Table 2.

**Table 2:** Polymers used for ICBM coating

RESOMER R 203 H (Boehringer Ingelheim), Lot.: 1038461, Poly (DL-lactide), inherent viscosity: 0.25–0.35 dl/g, mean molecular weight: 18–28 tsd Dalton.	
RESOMER RG 503 H (Boehringer Ingelheim), Lot.: 1037323, Poly (DL-lactide-coglycolide), acid terminated, lactide/glycolide ratio = 50:50, inherent viscosity: 0.32–0.44 dl/g, mean molecular weight: 24–38 tsd Dalton.	
RESOMER RG 504 H (Sigma Aldrich), Lot.: STBD1648V, Poly (DL-lactide-coglycolide), acid terminated, lactide/glycolide ratio = 50:50, inherent viscosity: 0.45–0.6 dl/g, mean molecular weight: 38–54 tsd Dalton.	
RESOMER RG 505 (Sigma Aldrich), Lot.: STBD7078V, Poly (DL-lactide-coglycolide), ester terminated, lactide/glycolide ratio = 50:50, inherent viscosity: 0.61–0.74 dl/g, mean molecular weight: 54–69 tsd Dalton.	
RESOMER L 206 S (Sigma Aldrich), Lot.: STBD8857V, Poly (DL-lactide), ester terminated, inherent viscosity: 0.8–1,2 dl/g, mean molecular weight: N/A.	

In order to gain probes with comparable inherent viscosity polymers with low inherent viscosity (i.e. RESOMER R203 H and RG503 H) were dissolved in 20% dichloromethane (DCM, Merck KGaA, Darmstadt) and polymers with high inherent viscosity (i.e. RESOMER RG504 H, RG505, L 206 S) in 10% DCM. 600 μl of these solutions were pipetted onto the ICBM accounting for 120 mg polymer per ICBM for RESOMER R203 H and RG503 H as well as 60 mg polymer per ICBM for RESOMER RG504 H, RG505 and L 206 S. Probes were stored at 4 °C. For analysis of polymer-coated ICBM morphology PLA- as well as PLGA-coatings were compared when dissolved in 5% DCM, ethyllactate (EthLac) and dimethyl sulfoxide (DMSO) using electron microscopy.

### Scanning electron microscopy (SEM)

SEM was conducted using the Hitachi S 3000 N microscope (Hitachi High-Technologies Canada, Inc., Toronto, Canada). Probes were prepared with critical point drying (CPD 030 Ball-Tec, Leica, Wetzlar, Germany) and gold coating (Cressington Sputter 108 Auto, Agar Scientific, Stansted, United Kingdom). To avoid damage of the polymer-coatings probes were critically point dried in an evacuated glass desiccator with silicate beads [22]. For SEM inspection probes were cut perpendicularly using a scalpel. 100x, 350x and 2500x magnification was used at a working distance of 18–19 mm.

### Release kinetics

Detection of FITC-BSA fluorescence values was conducted with TECAN GENios© Microplate reader and the software XFLUOR4 version 4.51. Samples were placed in black 96-well plates. Detection mode was set to „top” with fluorescence detection only occurring from the surface. Emission wavelength was 535 nm and excitation wavelength 485 nm. Target temperature was 29 °C. Preset mode was adjusted to three light pulses at 20 μs intervals at gain 57 per measurement.

Release kinetics of FITC-BSA from ICBM was measured in two groups to simulated possible in-vivo conditions of resorption. In one group ICBM were incubated in 24-well plates at 2 ml distilled water medium which was changed after each measurement (Table 3). In group two ICBM were incubated in 6-well plates at 6 ml distilled water medium which was not changed; the required 125 μl medium for measurement in GENios©, however, was refilled with distilled water (Table 4). Measurements were conducted after 6, 12 and 24 h (h) as well as after 3, 5, 7, 9 and 11 days (d). 125 μl volumes were used for the measurements.

**Table 3:** Mean amount of applied polymer per ICBM with medium change (MC)

Type of coating	Mean amount of coating in mg	Standard deviation [SD]
RESOMER R 203 H, MC	130.2	1.63
RESOMER RG503 H, MC	125.8	4.17
RESOMER RG504 H, MC	62.6	1.62
RESOMER RG505, MC	64.2	0.86
RESOMER L 206 S, MC	54.2	0.55

**Table 4:** Mean amount of applied polymer per ICBM without medium change (WMC)

Type of coating	Mean amount of coating in mg	Standard deviation [SD]
RESOMER R 203 H, WMC	127.6	1.17
RESOMER RG503 H, WMC	121.4	5.22
RESOMER RG504 H, WMC	62.2	1.21
RESOMER RG505, WMC	63.6	1.89
RESOMER L 206 S, WMC	55.3	0.74

### Statistics

A within subjects analysis of variance (ANOVA) was used to compare mean fluorescence values between the different polymer coatings (RESOMER R 203 H, RG503 H, RG 504 H, RG505, L 206 S) and control at each time of measurement (6 h, 12 h, 24 h, 3 d, 5 d, 7 d, 9 d, 11 d) for group 1 and 2. Significance level was set to *p* < 0.05. Bonferroni was used as the post-hoc test. Shapiro-Wilk normality test as well as Levene’s test for homogeneity of variance were used. Per time of measurement 6 samples were used for each polymer coating as well as control (*n* = 6). Statistics were analysed with SPSS version 24. For construction of tables Microsoft Excel© was used.

## Results

### Properties of ICBM

**Fig. 1:**
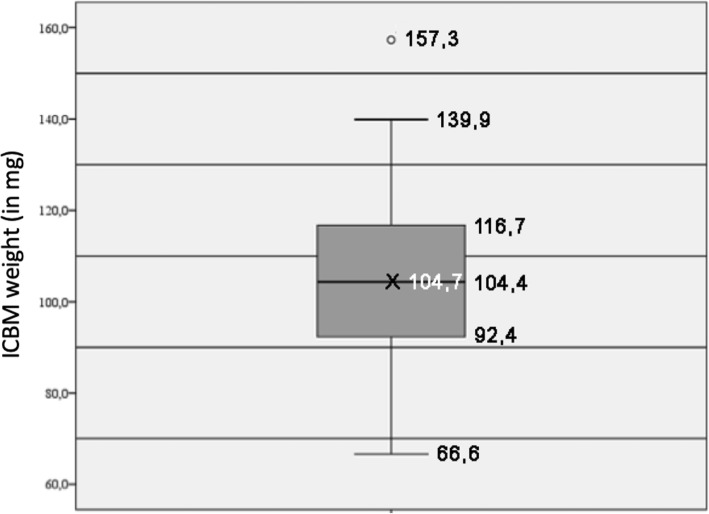
Boxplot for the weight distribution in milligrams of insoluble bovine collagenous bone matrices (ICBM). Mean weight was 104.7 mg and median weight 104.4 mg

**Fig. 2:**
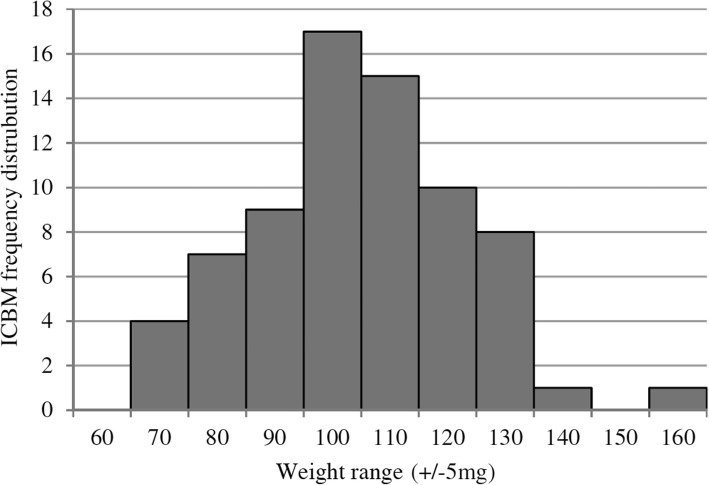
Frequency distribution of insoluble bovine collagenous bone matrices (ICBM) weight within weight ranges of ±5 mg

In total 72 ICBM probes were analysed. Mean weight of ICBM was 104.7 mg with a minimum weight of 66.6 mg and a maximum weight of 157.3 mg as well as a standard deviation [SD] of 17.46 mg (Fig. 1). Normal distribution was assessed with Kolmogorov-Smirnov and Shapiro-Wilk tests (Fig. 2). Tables 3 and 4 summarize the amount of applied polymer coating both for the group with and without medium change after weighing the ICBM before and after coating. Control groups of uncoated ICBM lost on average 3 mg of weight in the process of critical point drying in the form of water.

### Determination of the standard curve for fluorescence measurement of FITC-BSA

**Fig. 3:**
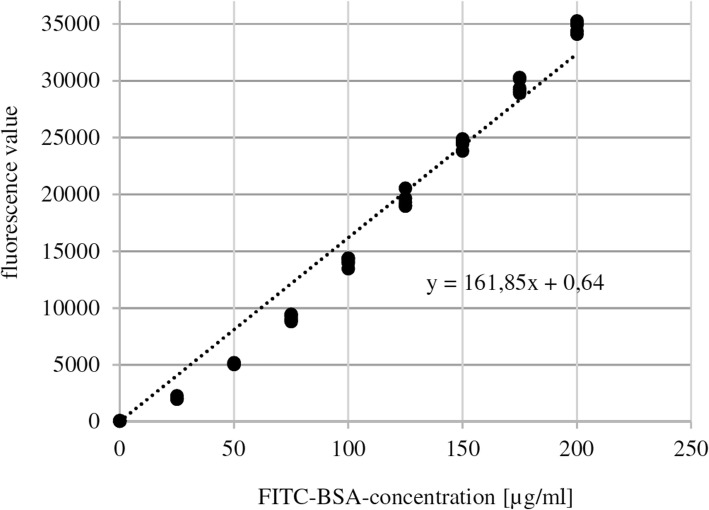
Dilution series of fluorescein isothiocyanate-labeled bovine serum albumin (FITC-BSA) and standard curve

Preliminary tests showed a linear correlation between concentrations of FITC-BSA and the measured fluorescence values detected by GENios© (Pearson: *r* = 0.993, *p* = 0.000). Figure 3 shows the standard curve which allows conversion of a measured fluorescence value into the respective FITC-BSA concentration (*y* = 161.85x + 0.64). Repeated measurements showed a mean decrease in fluorescence values of 2.5%.

### Release kinetics of FITC-BSA from ICBM with medium change

**Fig. 4:**
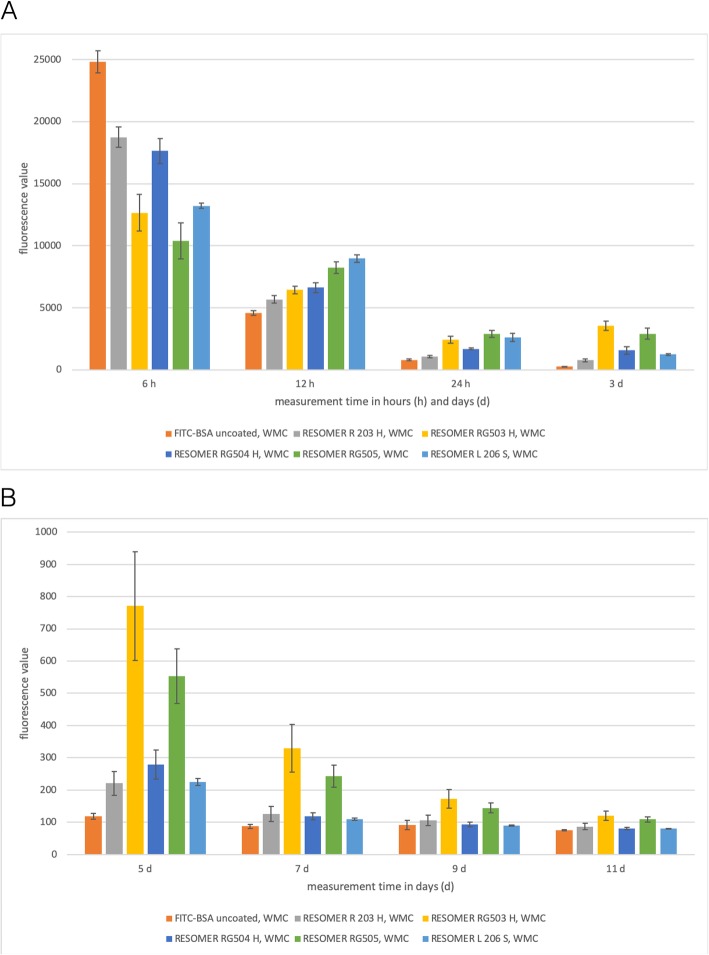
**a** Fluorescein isothiocyanate-labeled bovine serum albumin (FITC-BSA) release kinetics for measurement after 6 h (h), 12 h, 24 h and 3 days (d) in the group with medium change (WMC). Every bar symbolizes the mean fluorescence value measured for the respective measurement point. From left to right the bars display polymers with inherent viscosity ranked in ascending order. Data are expressed as means ± SEM of six measurements per time point. **b** Fluorescein isothiocyanate-labeled bovine serum albumin (FITC-BSA) release kinetics for measurement after 5 days (d), 7 d, 9 d and 11 d in the group with medium change (WMC). Every bar symbolizes the mean fluorescence value measured for the respective measurement point. From left to right the bars display polymers with inherent viscosity ranked in ascending order. Data are expressed as means ± SEM of six measurements per time point

Within the first 6 h release of FITC-BSA is highest in uncoated ICBM, whereas it is lower in coated ICBM with higher inherent viscosity. The amount of FITC-BSA released from uncoated ICBM within the first 6 h totals approximately 81% of the entire protein release within the observed 11 d and approximately 62% of the initial load of 500 μg (Fig. 4a and b). RESOMER L 206 S released 50% of its entire protein release within the first 6 h which equals 33% of the initial 500 μg FITC-BSA load (Fig. 4a and b). At the 12 h measurement the relations have partly turned upside down with RESOMER L 206 S releasing the highest amount of FITC-BSA with 34%, whereas the uncoated ICBM only released 15%. At the measurements points 24 h, 3 d and 5 d coated polymers with medium inherent viscosity levels like RESOMER RG 503 and RESOMER 505 showed the highest release rates (Fig. 4a and b). After 5 d, 7 d, 9 d and 11 d only very minimal amount of FITC-BSA were released into the medium.

**Fig. 5:**
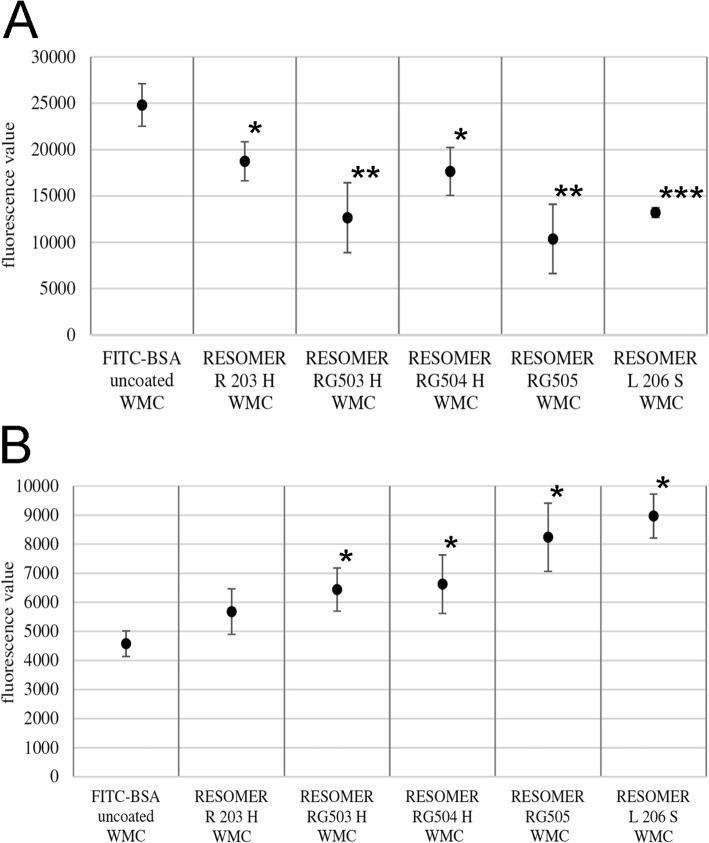
Analysis of mean fluorescence values of released FITC-BSA from polymer-coated ICBM in the group with medium change (WMC). **a** FITC-BSA release in the WMC group after 6 h (h), **b**) FITC-BSA release in the WMC group after 12 h (h). Data are expressed as mean ± STD of six measurement repeats per time point. One Way ANOVA, *p < 0.05, **p < 0.01, ***p < 0.001 as compared to FITC-BSA uncoated control

Figure 5a displays the fluorescence values of FITC-BSA released from the different polymer-coated ICBM into the medium after 6 h. RESOMER R 203 H, RESOMER RG503 H, RESOMER RG504 H, RESOMER RG505 and RESOMER L 206 S release significantly less FITC-BSA into the medium than uncoated ICBM (*p* < 0.05) (Fig. 5a). No significant differences in released FITC-BSA are found between RESOMER R 203 H and RESOMER RG504 H (*p* > 1.000) and RESOMER RG503 H, RESOMER RG505 and RESOMER L 206 S (*p* > 1.000).

At the 12 h measurement point RESOMER RG503 H, RESOMER RG504 H, RESOMER RG505 and RESOMER L 206 S released significantly more FITC-BSA into the medium compared to uncoated ICBM (Fig. 5b). Only RESOMER R 203 H did not show a significantly elevated FITC-BSA release (*p* = 0.37). Until measurement point 5 d RESOMER RG 503 showed the highest release rate of FITC-BSA and until measurement point 11 d was the only one with significantly higher FITC-BSA release rate compared to uncoated ICBM (*p* = 0.007). However, between 9 d and 11 d the released FITC-BSA amount was as small as 0.7 μg.

### Release kinetics of FITC-BSA from ICBM without medium change

**Fig. 6:**
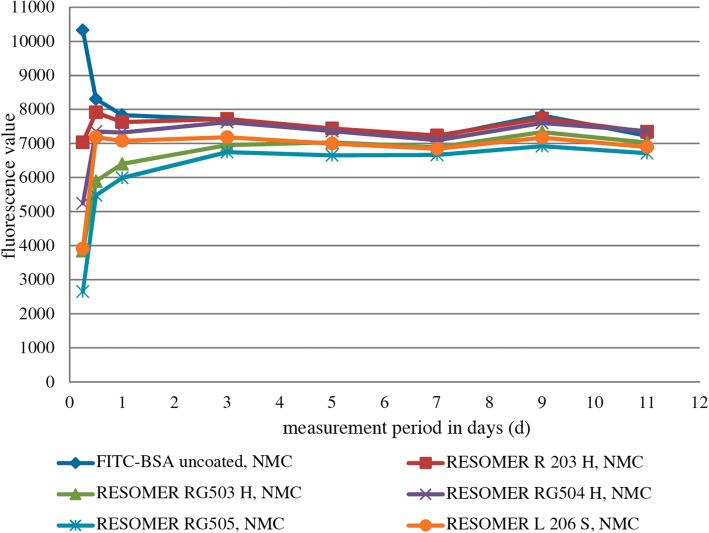
Fluorescein isothiocyanate-labeled bovine serum albumin (FITC-BSA) release kinetics for every measurement point in the group without medium change (NMC). Every bar symbolizes the mean fluorescence value measured for the respective measurement point

**Fig. 7:**
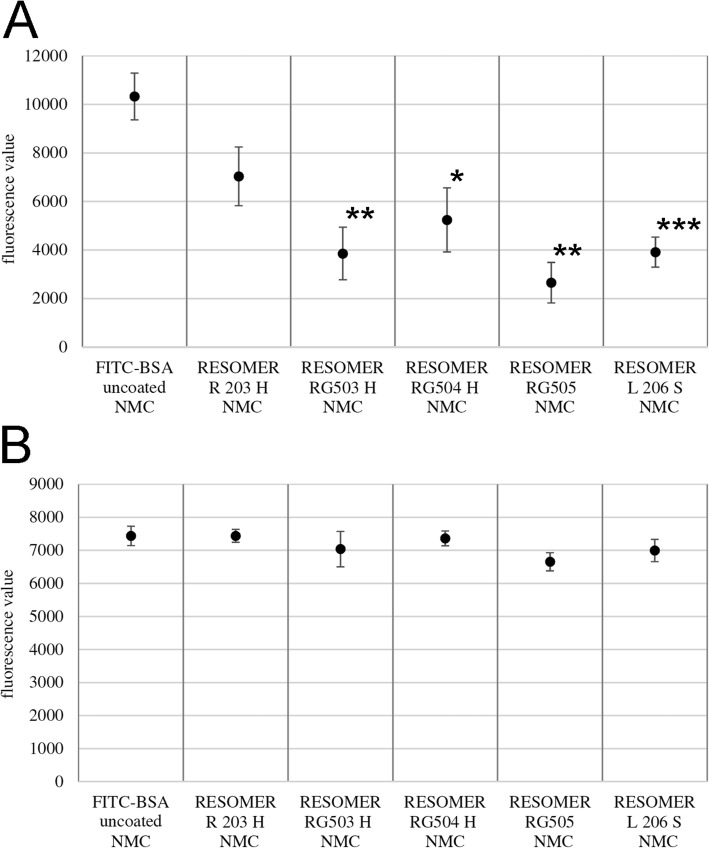
Analysis of mean fluorescence values of released FITC-BSA from polymer-coated ICBM in the group without medium change (NMC). **a** FITC-BSA release in the NMC group after 6 h (h), **b**) FITC-BSA release in the NMC group after 5 days (d). Data are expressed as mean ± STD of six measurement repeats per time point. One Way ANOVA, *p < 0.05, **p < 0.01, ***p < 0.001 as compared to FITC-BSA uncoated control

Differences in the amount of released FITC-BSA were most obvious within the first 3 d in the group without medium change (Fig. 6). RESOMER R 203 H, RESOMER RG503 H, RESOMER RG504 H, RESOMER RG505 and RESOMER L 206 S all showed an initial burst in FITC-BSA release within the first 24 h followed by drop into a saturation range until 11 d (Fig. 6). Uncoated ICBM, however, showed a continuous decline FITC-BSA release. Figure 7a displays the fluorescence values of FITC-BSA released from the different polymer-coated ICBM into the medium after 6 h. The mean of the quotient of the fluorescence values of FITC-BSA with and without medium change is 3.16. Hence, the amount of FITC-BSA released in the group without medium change on average is 5% higher compared to the group with medium change. After 12 h in the group with medium change FITC-BSA release was highest when molecular weight of polymer-coating was higher. In the group without medium change RESOMER 206 S, after 12 h, also showed the highest amount of FITC-BSA release. After 24 h only RESOMER RG 503 (*p* = 0.005) and RESOMER RG 505 (*p* < 0.001) showed less FITC-BSA release than the uncoated control. At measurement point 5 d only RESOMER RG 505 shows a smaller amount of released FITC-BSA than control (Fig. 7b).

### Morphologic differences in polymer-coated ICBM in SEM

**Fig. 8:**
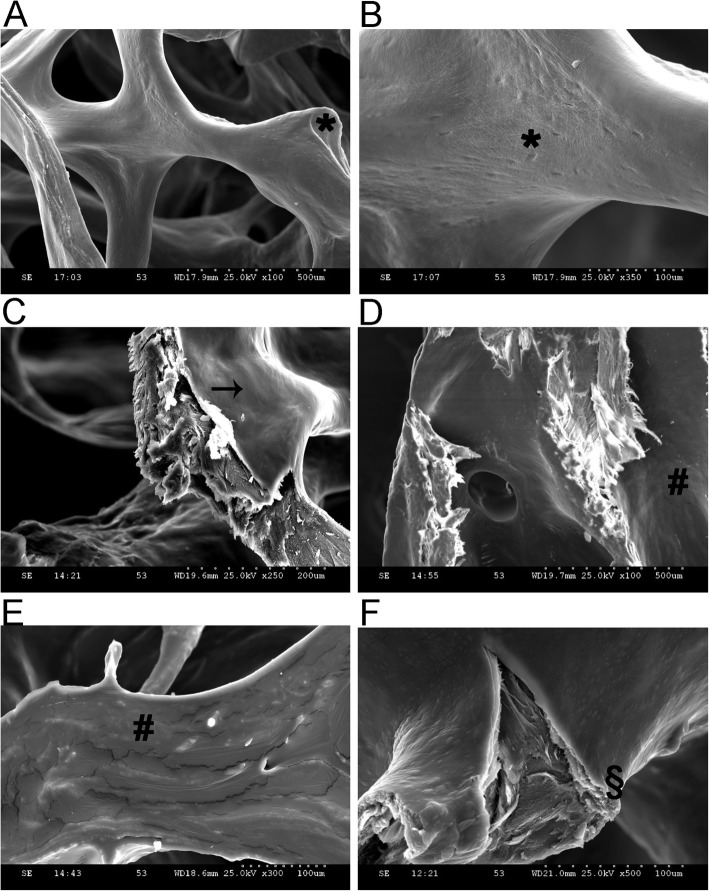
Micrographs of insoluble bovine collagenous bone matrices (ICBM) in scanning electron microscopy. **a** Uncoated ICBM at 100x magnification; * troughs and indentations, **b**) Uncoated ICBM at 350x magnification; * troughs and indentations, **c**) ICBM coated with 10% PLA (polylactic acid) in DCM (dichloromethane) solvent agent at 250x magnification; → homogenous layer, **d**) ICBM coated with 5% PLGA (poly-lactic-glycolic-acid) in EthLac (ethyllactate) solvent agent at 100x magnification; # bumpy layer, **e**) ICBM coated with 5% PLGA in EthLac solvent agent at 300x magnification; # bumpy layer, **f**) ICBM coated with 5% PLGA in DMSO (dimethyl sulfoxide) solvent agent at 500x magnification; § break-off edges

None of the native ICBM showed remnants of cancellous bone other than the collagen type I matrices. Porous collagen type I structures showed diameters between 50 and 700 μm linked to natural fluctuations of the material. Untreated ICBM showed troughs and indentations on a coarse surface in 100x and 350x magnification images (Fig. 8a and b).

**Fig. 9:**
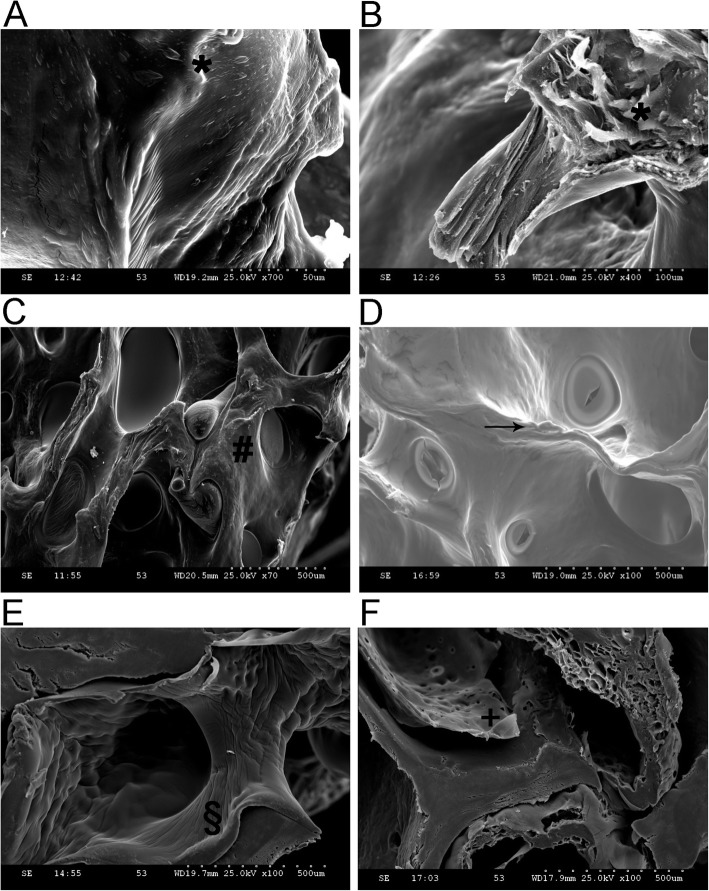
Micrographs of insoluble bovine collagenous bone matrices (ICBM) in scanning electron microscopy. **a** ICBM coated with 5% PLGA in DMSO (dimethyl sulfoxide) solvent agent at 700x magnification; * evenly distributed glassy polymer distensions, **b**) ICBM coated with 5% PLGA in DMSO solvent agent at 400x magnification; * evenly distributed glassy polymer distensions, **c**) ICBM coated with 20% PLGA (poly-lactic-glycolic-acid) in DCM (dichloromethane) solvent agent at 70x magnification; # homogenous layer, **d**) ICBM coated with 5% PLGA in DCM solvent agent at 100x magnification; → cracks, **e**) ICBM coated with 20% PLGA in DCM solvent agent and FITC-BSA labeling at 100x magnification; § wave-like distensions, **f**) ICBM coated with 20% PLGA in DCM solvent agent after incubation in distilled water for 3 days (d) at 100x magnification; + holes and indentations

RESOMER R 203 H, RESOMER RG503 H, RESOMER RG504 H, RESOMER RG505 and RESOMER L 206 S were all best soluble in DCM. At concentrations of 5–10% the polymer-coatings sat evenly on the ICBM. Figure 8c shows an ICBM PLA 10% coating with DCM at 250x magnification. By contrast, coatings with EthLac as the solvent did not show a homogenous but bumpy layer on the ICBM (Fig. 8d and e). Polymer coatings in DMSO show evenly distributed glassy distensions with a diameter between 7 and 10 μm (Fig. 9a and b). The inner ICBM structure is fibrous at the break-off edges similar to EthLac (Fig. 8f).

As preliminary tests showed that thin polymer-coatings did not significantly reduce FITC-BSA release ICBM were coated with thicker polymer-layers. Whereas PLGA 5% coatings in DCM developed cracks under the exposure of the electron beam of SEM (Fig. 9d) PLGA 20% coatings in DCM did not show such effects (Fig. 9c).

ICBM labelled with FITC-BSA and coated with a polymer showed wavelike distensions along the surface (Fig. 9e). Gap formation was observed between coating and ICBM. After 3 d incubation in distilled water erosion and degradation processes of the polymer became obvious. The previously smooth surface but also the inner structure of the polymer layer showed holes and indentations resulting in shedding of the polymer layer off the ICBM (Fig. 9f). The labelled FITC-BSA was not visible in SEM. Furthermore, no differentiation between PLA and PLGA coatings could be determined.

## Discussion

The aim of modern tissue engineering is to facilitate tissue regeneration by means of an ideal combination of cytokines and drug carriers. Maximum efficiency of a cytokine can only be achieved when it is released early on and continuously from its carrier. The drug carrier itself should maintain volume stability to enable incorporation into the surrounding tissue [9]. Hence, it is unfavorable to synthesize the drug carrier from biodegradable material as cytokine release would be limited in time until the carrier is degraded or lost its structural integridy. Therefore, we used PLA and PLGA as separate polymer-coatings of the ICBM. It is expected that polymer-coated ICBM are incorporated into the surrounding tissue in the same way native ICBM are. This is the first time the model protein FITC-BSA has been bound to PLA and PLGA coatings to assess its release throughout polymer degradation. It has been shown that PLA, PLGA and ICBM are biocompatible with no signs of exuberant immune responses [23, 24]. Furthermore, PLA and PLGA are believed to foster osteogenic differentiation of stem cells [25]. The model protein FITC-BSA used in this study is comparable to bone morphogenetic protein-2 (BMP-2) in terms of molecular size and form connecting the results of this study to physiological processes [26]. Also, the biological binding affinity of BSA and BMP-2 to collagen type I are reported to be similar leading to related release kinetics [27, 28]. A series of dilutions generated the standard curve for FITC-BSA which showed a linear correlation of fluorescence and protein concentration. We tested that there was no interaction between the ICBM as well as the polymer-coatings and the fluorescence signal. The fluorescence of FITC-BSA was stable for 2 weeks when kept at 4 °C, in aqueous solution in a dark container. However, it must be taken into consideration that detected fluorescence values are not to be equated with detection of a biologically active protein. It is conceivable that despite BSA degradation FITC fluorescence is still detected leading to false-positive results. For BSA not fulfilling a function which could be detected in-vitro analyses of its secondary structure besides the primary structure could demonstrate whether BSA is still intact. Determan et al. (2004) found with electrophoresis that the protein structure of a small fraction of BSA disintegrated over the course of release analysis [29]. In their study, the amount and velocity of BSA degradation was time, temperature and pH-dependent. For this study the results of Determan et al. (2004) imply that false-positive BSA detection could have been aggravated over time and decline of the pH-value throughout the course of the measurement time. Therefore, in further studies the degree of BSA degradation should be tested as well.

In SEM we compared the solvents DCM, EthLac and DMSO. DCM was the only solvent which lead to a homogeneous attachment of polymers to ICBM, whereas EthLac and DMSO caused inhomogeneous surface structures. These results are in accordance with the findings of Madsen et al. (2015) who compared the solvents DMC, acetone and ethanol [30]. It was shown that protein release through a polymer-coating was slower the smaller the differences in solubility between solvent and polymer are. Hence, the differences in SEM are most likely not only morphologically relevant but have influence on the release kinetics of FITC-BSA.

Even though general ossification time of critical size bony defects is weeks to months we analyzed a period of merely 11 d since initiation of bone regeneration through BMP-2 was demonstrated in early regeneration phase [31]. FITC-BSA release from uncoated ICBM is characterized by an initial protein overshoot within the first 12 h and a steady decline until the third day. From day three onward the release rate reaches a plateau with almost no relevant amounts of FITC-BSA release. These results are consistent with other studies analyzing the release of growth factors from uncoated collagen matrices [32, 33]. It seems plausible that FITC-BSA is insufficiently bound to native ICBM since 81% protein release was observed within the first 6 h. However, the absolute amount of protein release measured by the fluorescence values within the solvent agent is only 70% in the group with medium change and 50% in the group without medium change. In can be deduced that ether a certain fraction of FITC-BSA is bound to ICBM or released FITC-BSA bound to the surface of the reaction tube. To prevent FITC-BSA adsorption to the reaction tube Determan et al. (2004) applied 3% sodium lauryl sulfate as surfactant to the solvent medium [29]. Binding of BSA to ICBM is non-covalent, through van der Waals forces, ionic bonds as well as hydrophobic and electrostatic interactions [34]. Serro et al. calculated BSA monolayer saturation to be 4 mg/m^2^ [35]. It is likely that the initial FITC-BSA release is due to an overload of protein that cannot bind to ICBM [36]. Nevertheless, the precocious FITC-BSA release from native ICBM is unsatisfactory given that growth factors like BMP-2 would culminate in an unphysiological way and could cause the known side effects.

This study could show an influence of polymer size and amount of PLA and PLGA on the release kinetics of FITC-BSA. It was seen that in early stages FITC-BSA release was smaller the higher polymer concentration was and the bigger its molecule size was. The influence of polymer inherent viscosity on FITC-BSA release is higher than polymer concentration. No differences in protein release were seen between PLA and PLGA. Contemporary studies showed that burst-release of proteins coupled to polymer matrices is common [37]. To prevent early protein wash-out with delamination of the polymer from the ICBM as observed in SEM images in this study covalent bounds of protein and polymer layer seem worth investigation. Whereas binding of FITC-BSA to ICBM directly poses the disadvantage of ICBM degradation-dependent FITC-BSA release coupling of FITC-BSA to a polymer-coating offers protein accumulation in a sequestered form in the extracellular compartment where intercellular communication processes often require a protein-receptor-complex [38]. Gharibjanian et al. (2009) showed that covalent bound BMP-2 to PLGA and PCL increased markers of osteogenic differentiation in pre-osteoblasts in mice compared to control [39].

## Conclusions

This study shows a decrease in FITC-BSA release, especially from medium-size polymer coatings. Morphologic structure of PLA and PLGA coatings in SEM differ among different solvent agents. This leads to changes in the attachment of polymer coating and ICBM resulting in different FITC-BSA release rates. These results highlight the complex release process of proteins (i.e. growth factors, cytokines) from drug carrier scaffolds. Medium size polymer coatings may prove to be ideal for drug carrier materials.

## Data Availability

The datasets used and/or analysed during the current study are available from the corresponding author on reasonable request.

## Abbreviation

ANOVA: Analysis of variance
BMP-2: Bone morphogenetic protein-2
BSA: Bovine serum albumine
DMC: Dichloromethane
DMSO: Dimethyl sulfoxide
EthLac: Ethyllactate
FDA: Federal Drug Agency
FITC-BSA: Fluorescein isothiocyanate-labeled bovine serum albumin
ICBM: Insoluble bovine collagenous bone matrices
IL-1: Interleukin-1
IL-6: Interleukin-6
PLA: Polylactic acid
PLGA: Poly-lactic-glycolic-acid
rhBMP-2: Recombinant bone morphogenetic protein-2
SD: Standard deviation
SEM: Standard error of the mean
TNF-α: Tumor necrosis factor-α
